# Colo-Salpingeal Fistula and Secondary Tubo-Ovarian Abscess as Rare Complications of Sigmoid Diverticulitis: A Case Report

**DOI:** 10.7759/cureus.109640

**Published:** 2026-05-25

**Authors:** Caden Fuller, Nadim Bou Zgheib, John Diks

**Affiliations:** 1 Pathology and Laboratory Medicine, West Virginia School of Osteopathic Medicine, Lewisburg, USA; 2 Obstetrics and Gynecology, Marshall University, Huntington, USA; 3 Pathology, Marshall University, Joan C. Edwards School of Medicine, Huntington, USA

**Keywords:** colo-salpingeal fistula, complications, rare, secondary tubo-ovarian abscess, sigmoid diverticulitis

## Abstract

Secondary tubo-ovarian abscess (TOA) arising from gastrointestinal pathology is a rare and diagnostically challenging condition. We report the case of a 43-year-old gravida one para one woman with a chronic pelvic abscess refractory to prolonged antibiotic therapy and multiple percutaneous drainage attempts. The patient presented with persistent lower abdominal pain, low-grade fever, and decreased output from a previously placed pelvic drain. Imaging demonstrated a complex left pelvic mass with subsequent concern for rectosigmoid colon involvement. Surgical management consisted of robotic total laparoscopic hysterectomy, bilateral salpingectomy, left salpingo-oophorectomy, extensive lysis of adhesions, and robotic-assisted sigmoid colon resection with primary anastomosis. Final pathology confirmed colonic diverticulitis with a colo-salpingeal fistula and secondary tubo-ovarian abscess. This case highlights the importance of recognizing secondary TOA as a potential complication of diverticular disease and underscores the role of multidisciplinary surgical management when conservative therapy fails.

## Introduction

Tubo-ovarian abscess (TOA) is a severe complication of pelvic inflammatory disease and is traditionally attributed to ascending gynecologic infection [1]. However, in rare cases, TOA may arise secondary to adjacent gastrointestinal pathology, such as sigmoid diverticulitis [1,2]. In such cases, chronic inflammation may result in fistulous communication between the bowel and adnexa, leading to secondary infection of the tubo-ovarian complex.

Primary TOA is often managed conservatively with antibiotics and percutaneous drainage; however, a subset of patients develops chronic, refractory TOAs that necessitate surgical intervention. Although rare, colo-salpingeal fistulas represent a significant challenge in diagnosis and management, often mimicking other conditions such as primary tubo-ovarian abscesses or even ovarian neoplasms [1,2].

Colonic diverticulosis is a highly prevalent condition in Western countries, affecting over 50% of individuals over the age of 50, with 10-25% of the population experiencing symptomatic diverticular disease in their lifetime [1,3,4]. While the most common complications of acute diverticulitis include perforation, intestinal obstruction, and pericolic abscess formation, fistula formation occurs in approximately 10-20% of patients [3,4]. These tracts most frequently manifest as colo-vesical, colo-vaginal, or involve the small bowel, whereas colo-uterine fistulae are exceptionally rare due to the protective thickness of the uterine myometrium [1]. Furthermore, communication between the colon and the adnexa typically arises in the context of primary ovarian neoplasms, primary ovarian abscesses, or Crohn’s disease, making colo-ovarian fistulas secondary to acute colonic diverticulitis an uncommon entity with few reported cases [3]. Even more unusual are colosalpingeal fistulas (only 2% of cases), which represent an exceedingly rare complication of sigmoid diverticulitis, with a review of the literature revealing fewer than roughly 15 cases characterizing this specific pathology [4-6]. Colo-salpingeal fistulas pose significant diagnostic challenges, as their presentation can mimic primary gynecologic disease. Imaging findings may be equivocal, and fistulous tracts are not always readily apparent on initial studies. Failure to recognize a gastrointestinal source can delay definitive management and prolong morbidity.

We present a case of a 43-year-old woman with sigmoid diverticulitis complicated by a colo-salpingeal fistula and secondary tubo-ovarian abscess that proved refractory to multiple conservative treatment attempts. The patient's clinical course, diagnostic workup, and successful surgical management, including robotic-assisted sigmoid colon resection, are detailed. This case underscores the importance of considering gastrointestinal etiologies in chronic TOAs and highlights the role of comprehensive surgical strategies in achieving definitive resolution, especially when conservative approaches prove ineffective.

## Case presentation

A 43-year-old primipara woman was transferred from an outside hospital for management of a chronic pelvic abscess. She presented with lower abdominal pain and decreased output from a previously placed pelvic drain. Her obstetric history was notable for one prior cesarean delivery. She denied a history of sexually transmitted infections, new sexual partners, abnormal vaginal discharge, or malodor. The patient did not desire future fertility and was amenable to hysterectomy if required. Her clinical course was notable for multiple prior hospitalizations over several months for a persistent pelvic abscess, with concern for tubo-ovarian abscess, diverticulitis, and a possible peridiverticular abscess. She had undergone at least four image-guided percutaneous drain placements without resolution. Prior abscess cultures grew *Escherichia coli* and *Klebsiella* species, both of which were sensitive to amoxicillin-clavulanate, which she was receiving at the time of presentation. 

On examination, she was hemodynamically stable with a low-grade fever. Abdominal examination revealed a soft abdomen with tenderness in the lower quadrants without rebound or guarding. A pelvic drain was present with minimal output. Laboratory evaluation demonstrated mild normocytic anemia and hypoalbuminemia without leukocytosis. Early imaging during her disease course demonstrated a complex left pelvic abscess without definitive evidence of fistulous communication. A contrast enema study failed to identify a connection between the sigmoid colon and the pelvic abscess. However, later in the clinical course, contrast-enhanced computed tomography demonstrated a persistent left-sided tubo-ovarian abscess with a fistulous connection to the sigmoid colon.

**Figure 1:**
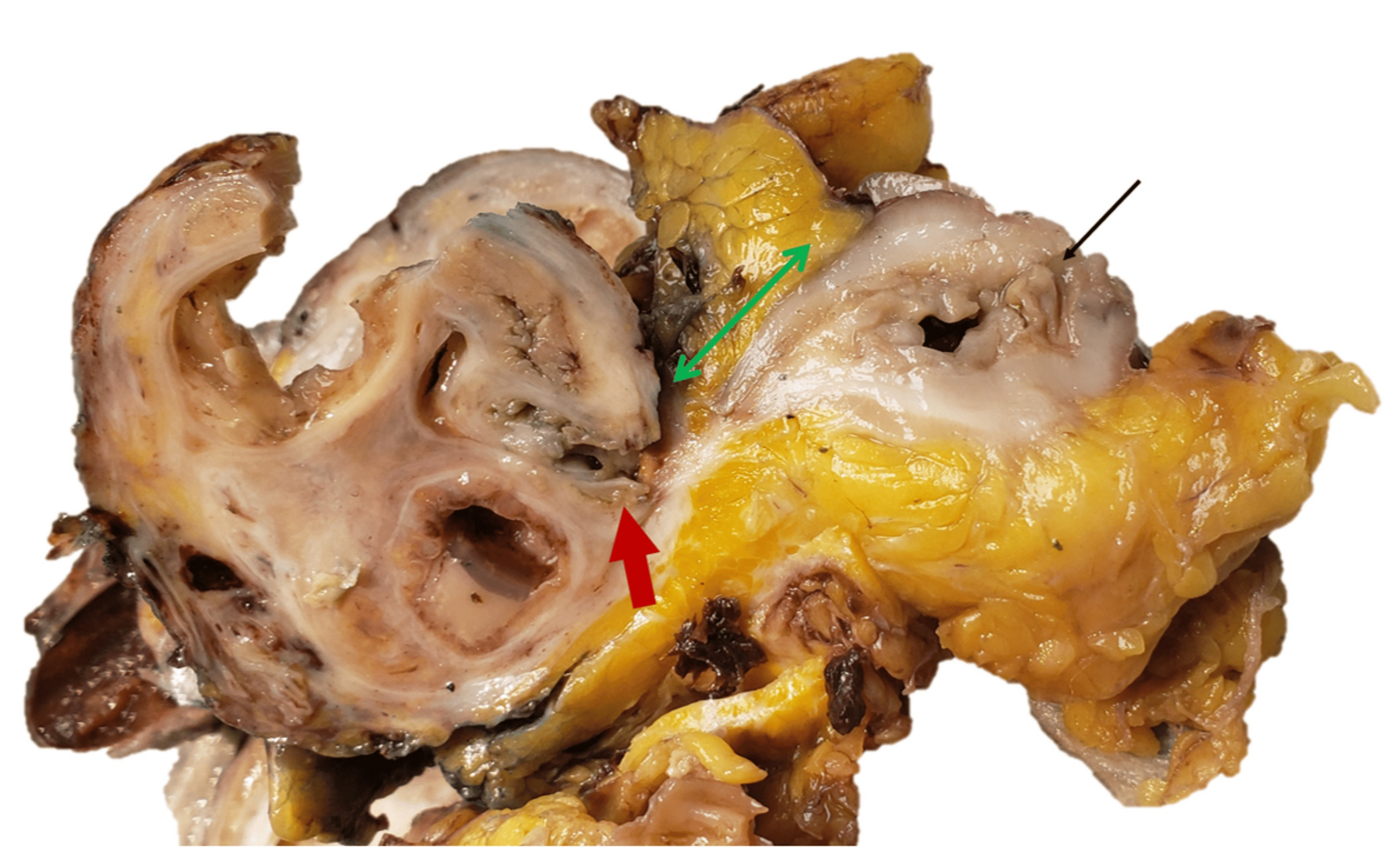
Gross picture of the left adnexal mass and sigmoid colon showing diverticulosis (red arrow), fistula track (green double-headed arrow), and left fallopian tube (black arrow).

Given the chronicity of the disease, failure of conservative management, and concern for bowel involvement, a multidisciplinary surgical approach was pursued. The patient underwent examination under anesthesia, including diagnostic laparoscopy, robotic total laparoscopic hysterectomy with bilateral salpingectomy, left salpingo-oophorectomy, extensive lysis of adhesions and enterolysis, robotic-assisted sigmoid colon resection with primary anastomosis, and cystoscopy with temporary ureteral stent placement. Intraoperatively, a 10-centimeter complex left ovarian mass was identified with dense adhesions and extensive fibrosis involving the left pelvic sidewall, retroperitoneum, and rectosigmoid colon. The planes between the adnexal mass and sigmoid colon were inseparable (Figure 1). Therefore, the mass and affected segments of the sigmoid colon were removed en bloc. Estimated blood loss was two hundred fifty milliliters, and no intraoperative complications occurred.

**Figure 2:**
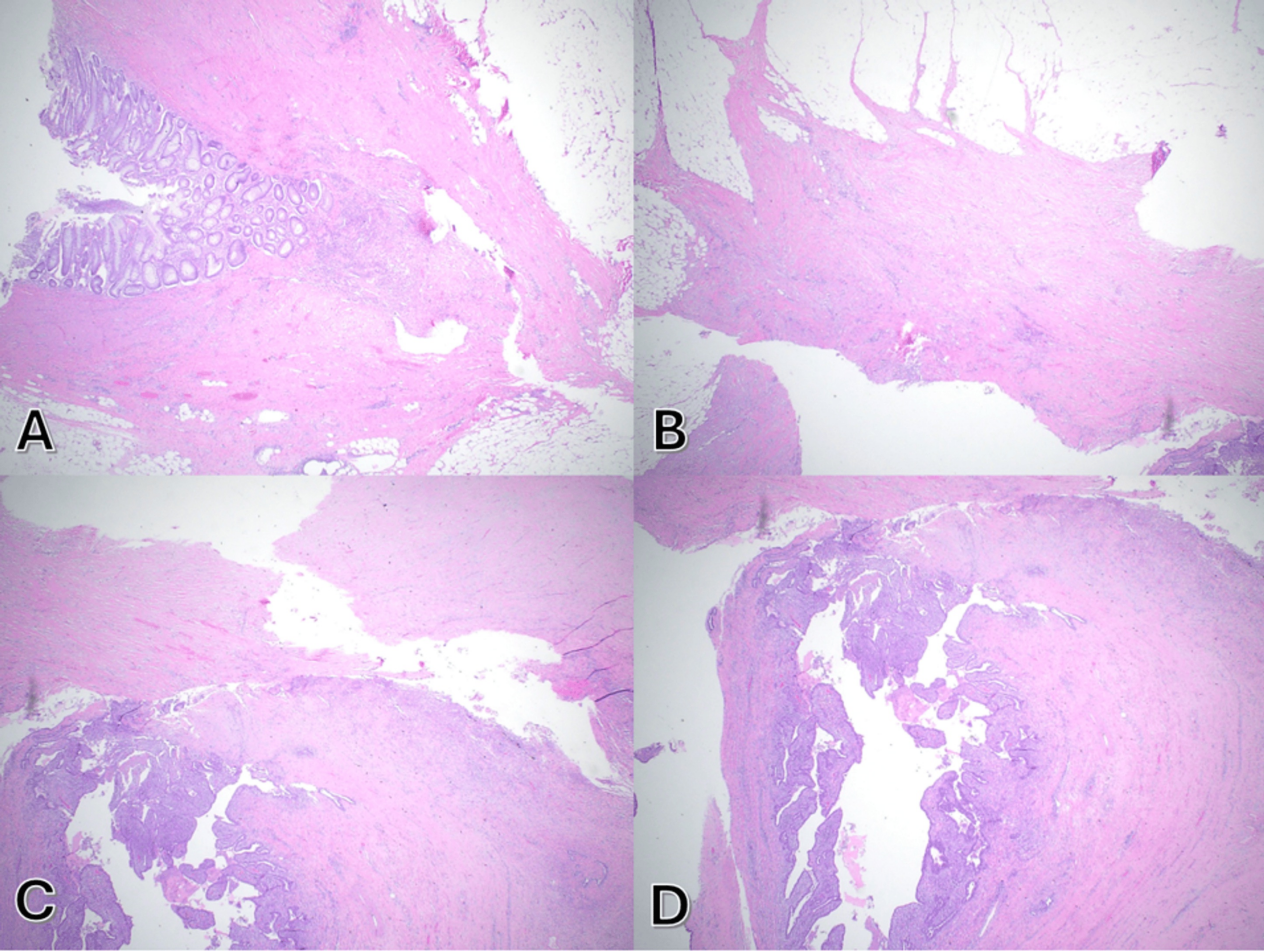
H&E sections (200x) showing (A) sigmoid colon with diverticulosis and diverticulitis and (B)&(C) fistula tract, (D) tubulo-ovarian abscess.

At two-week postoperative follow-up, the patient was recovering well without reported complications. Final pathology revealed colonic diverticulosis with diverticulitis, a colo-salpingeal fistula, and secondary tubo-ovarian abscess without evidence of malignancy (Figure 2). The patient was advised to continue postoperative restrictions and return for further follow-up as scheduled.

## Discussion

This case highlights a complex presentation of a secondary tubo-ovarian abscess (TOA) arising from sigmoid diverticular disease with fistulization to the adnexa, ultimately requiring definitive surgical management. Although TOA is classically associated with pelvic inflammatory disease, secondary adnexal involvement can occur when colonic inflammation or perforation extends into the fallopian tube and ovary, producing an inflammatory mass that mimics a primary gynecologic process [1]. Similar presentations have been described in the literature, where diverticulitis-related fistulas involving the female genital tract resulted in adnexal abscess formation rather than classic urologic or vaginal fistulas [2].

Colo-salpingeal fistulas are very uncommon compared with other diverticular fistulas. Anthony et al. emphasize that diverticular fistulas most commonly present as colo-vesical and colo-vaginal connections, with genital tract fistulas being much rarer [1]. Gopalan et al. describe fistula formation between the sigmoid colon and adnexa as an exceptional rarity, noting that these cases are frequently misdiagnosed initially as tubo-ovarian abscesses based on clinical presentation and early imaging findings, a pattern similarly reported in broader reviews of unusual diverticulitis-associated fistulas involving the female genital tract [2]. Additional literature confirms that communication between the colon and ovary or fallopian tube is rarely reported, despite the relative frequency of other diverticular fistulas [3].

Diagnosis can be challenging because conventional imaging may fail to clearly demonstrate a fistulous tract, particularly early in the disease course. Anthony et al. describe how CT imaging may fail to directly visualize fistulous tracts, even when secondary inflammatory changes are present [1]. In reported cases of colo-salpingeal fistulas (a subset of colo-adnexal fistulas), imaging findings may initially suggest a primary gynecologic abscess, with bowel involvement only becoming apparent on repeat imaging or intraoperatively [2]. Prior reports have demonstrated that colo-salpingeal fistulas may remain radiographically occult even on contrast-enhanced CT or MRI, with fistulous communication often not definitively identified until surgical exploration or pathologic examination [3]. These limitations underscore that negative imaging does not exclude fistulization when clinical suspicion remains high.

Several imaging and operative clues may aid diagnosis in complex cases. Gas within an adnexal collection has been highlighted as an important radiologic sign suggesting enteric communication, even when a discrete fistulous tract is not visualized [3,4]. Jangam and Gillespie describe cases in which adnexal gas and inflammatory collections were initially misinterpreted as gynecologic infections, with definitive diagnosis achieved only after surgical exploration [4]. Broader reviews of colo-salpingeal fistulas similarly emphasize that imaging sensitivity is difficult to establish due to the rarity of the condition and that diagnosis often relies on intraoperative and pathologic confirmation [5].

When conservative management fails, including prolonged antibiotic therapy and repeated percutaneous drainage, surgical intervention is typically required. Prior reports frequently describe en bloc resection of the involved bowel segment and affected adnexa as the definitive treatment strategy in fistulizing diverticular disease [1,4]. Jangam and Gillespie specifically state that resection of the sigmoid colon with the involved fallopian tube or ovary represents the mainstay of treatment for colo-salpingeal fistulas [4]. Reviews of similar cases recommend primary anastomosis in stable patients, reserving diversion for those with severe contamination or hemodynamic instability [5]. In the present case, the robotic platform facilitated meticulous dissection in a hostile surgical field characterized by dense adhesions and retroperitoneal fibrosis, allowing safe en bloc resection and reconstruction.

The precise incidence of tubo-ovarian abscess secondary to colonic diverticulitis with colo-salpingeal fistula formation is unknown, as this entity is exceedingly rare and described primarily in isolated case reports [6]. However, diverticulitis develops in approximately 20% of patients with colonic diverticulosis, and fistula formation is an established complication occurring in roughly 2% of diverticular disease cases [7]. Among fistulizing complications, colovesical and colovaginal fistulas are most common, while involvement of gynecologic adnexal structures such as the fallopian tube represents a very small minority of reported cases [7-9].

In summary, this case illustrates a rare presentation of sigmoid diverticulitis complicated by a colo-salpingeal fistula and secondary tubo-ovarian abscess. Persistent or recurrent tubo-ovarian abscesses that fail conservative management should raise suspicion for an underlying gastrointestinal source. In such cases, imaging findings may be inconclusive, and definitive diagnosis and treatment often require multidisciplinary surgical intervention with en bloc resection to achieve resolution [1-5].

## Conclusions

Secondary tubo-ovarian abscess due to sigmoid diverticulitis is a rare but important diagnostic consideration in patients with chronic or refractory pelvic abscesses. Early imaging may fail to identify fistulous communication, and a high index of suspicion is required. When conservative management fails, comprehensive surgical intervention, including en bloc resection of involved adnexa and bowel, can result in definitive resolution. This case highlights the importance of multidisciplinary collaboration and the utility of robotic-assisted surgery in managing complex pelvic inflammatory disease.
